# Revisiting the empirical case against perceptual modularity

**DOI:** 10.3389/fpsyg.2015.01676

**Published:** 2015-11-04

**Authors:** Farid Masrour, Gregory Nirshberg, Michael Schon, Jason Leardi, Emily Barrett

**Affiliations:** Department of Philosophy, University of Wisconsin-Madison, Madison, WI, USA

**Keywords:** perception, modularity, encapsulation, cognitive penetration, vision disorders, psychophysics

## Abstract

Some theorists hold that the human perceptual system has a component that receives input only from units lower in the perceptual hierarchy. This thesis, that we shall here refer to as the *encapsulation thesis*, has been at the center of a continuing debate for the past few decades. Those who deny the encapsulation thesis often rely on the large body of psychological findings that allegedly suggest that perception is influenced by factors such as the beliefs, desires, goals, and the expectations of the perceiver. Proponents of the encapsulation thesis, however, often argue that, when correctly interpreted, these psychological findings are compatible with the thesis. In our view, the debate over the significance and the correct interpretation of these psychological findings has reached an impasse. We hold that this impasse is due to the methodological limitations over psychophysical experiments, and it is very unlikely that such experiments, on their own, could yield results that would settle the debate. After defending this claim, we argue that integrating data from cognitive neuroscience resolves the debate in favor of those who deny the encapsulation thesis.

## Introduction

Participants in the debate over whether cognition can influence perception could be roughly divided into two camps. One camp consists of those who emphasize that the information that is received through the senses is not sufficient to uniquely determine the correct hypothesis about its distal causes. Their proposal is that in order to solve this under-determination problem, the inputs of perception must be supplemented by more information, such as the background beliefs of the perceiver. Perceptual processes thus have access to central cognition and are susceptible to influence by cognition. The opposing camp, which we refer to as the modularists, acknowledges that the input to the perceptual system needs to be supplemented to solve the under-determination problem. Nevertheless, they hold that the additional information is localized to the perceptual module.

A central intuition behind the modularist approach is that evaluating the incoming stimulus in light of a large body of information is time consuming and costly. And since a well-designed perceptual system should enable fast responses to changing environmental conditions, it must lack access to the totality of information that a perceiver has. Imagine contemplating the possible visual tricks that someone might have played on you when it seems that a lion is about to attack you on a safari. Even if that sort of contemplation is something that *you* are prone to do, it is better if *your perceptual system* does not have this propensity, and one way to guarantee that it does not is to limit its access to what is directly relevant to its domain of input. This is one of the main insights behind Fodor’s original distinction between what he calls “input analyzers” and “central cognition.” Input analyzers are in the business of fast analysis of incoming data on the basis of a limited body of information, which is typically domain specific, innate, and localized. Central cognition, in contrast, is in the business of belief formation, problem solving, contemplation, etc., on the basis of information that is often domain general, learned, and non-localized.

Our focus in this paper will be on the empirical evidence pertaining to a thesis that is at the core of the modularity debate. This is the thesis that the human perceptual system has components that are informationally encapsulated. Relying on Fodor’s terminology, we call these components modules^1^. As a first approximation, the claim that a module is informationally encapsulated means that the processes within the module have access only to the contents of other processes within the module as well as input from earlier units in the perceptual hierarchy^2^. Throughout the paper, we refer to the thesis that the perceptual system has at least one informationally encapsulated module as the *generic encapsulation thesis*.

If the encapsulation thesis is correct, then the functioning of the visual module should be free from content-sensitive influence both from other modules and cognitive processes “up stream” in the visual hierarchy. However, there is a large body of research, starting in the mid-twentieth century and extending to the present date, which purports to establish that perception can be influenced by cognitive factors such as the stimulus’s meaning, its familiarity, its predictability, the context it appears in, the concepts the perceiver uses to categorize it, and more (for some recent findings and reviews, see Hansen et al., 2006; Levin and Banaji, 2006; Lupyan and Spivey, 2008; Lupyan et al., 2010; Macpherson, 2012; Lupyan and Ward, 2013; Stokes, 2013; For earlier findings on the topic, see Bruner, 1957, 1973; Rock, 1983; Goldstone, 1995; and the extensive review in Pylyshyn, 1999).

The vast majority of this research comes from psychophysical studies that track potential cognitive effects on perceptual performance^3^. Modularists rarely deny the effects that these studies purport to show. Rather, their main strategy has been to argue that the results of these studies can be interpreted in ways that are compatible with their preferred version of the encapsulation thesis. In fact, a quick survey of the history of the debate over the empirical evidence reveals a pattern in which anti-modularists produce new results which are subsequently explained away by the modularists^4^.

We agree with the modularists that the many of the existing psychophysical findings could be explained away. However, we disagree with the modularists that this indicates that the encapsulation thesis is correct. In our view, reflection on the general methodological limitations of psychophysical experiments shows that it is very unlikely that such experiments, on their own, could yield results that could not be explained away by the modularists. It is therefore not the truth of the thesis, but the nature of psychophysical methodology, that is the underlying cause of the impasse. Our first goal in this paper is to defend this claim. However, we do not think that one should be skeptical of the possibility of an empirical resolution to this debate. In fact, we believe that integrating data from cognitive neuroscience will very likely resolve the debate in favor of the anti-modularists. Defending this claim is the second goal of this paper.

We have defined the generic encapsulation thesis as the thesis that there is an informationally encapsulated component of the perceptual system, namely a module. However, for reasons to be discussed soon, this paper focuses on a much more specific variant of the generic encapsulation thesis. This variant, roughly characterized, holds that there is an informationally encapsulated component of the perceptual system that gives rise to access conscious representations.

Here is how we will proceed. In the next section, we further explicate the generic encapsulation thesis, distinguish among some of its interesting variants, and elaborate on the link between these variants and some broader theoretical issues surrounding the modularity debate. This discussion helps clarify and justify our choice for focusing on the specific version of the thesis in the rest of the paper. However, those readers who are interested in quickly getting to our main arguments can skip straight to the “Psychological Case Against Encapsulation” section which focuses on the psychophysical evidence. In that section, we identify three core demands that psychophysical studies must meet in order to establish the failure of encapsulation. We then argue that the nature of these demands make it very unlikely that purely psychophysical studies would be sufficient to reject the thesis. Our methodological conclusion is that the empirical approach to the debate has to draw on data from sources other than purely psychophysical studies. This is what we shall do in the “Neural Case Against Encapsulation” section where we draw on recent findings in neuroscience and argue that they militate against the version of the encapsulation thesis that we describe in the next section.

## The Encapsulation Thesis

Earlier we defined the generic encapsulation thesis as the thesis that there is an informationally encapsulated component of the perceptual system, namely a module. A module is informationally encapsulated in the sense that its processes have access only to the contents of other processes within the module and the information provided by earlier units in the perceptual hierarchy. As we use the term, processes are transitions from one set of contentful states to another. A contentful state is one that can be assessed for veridicality. Under this definition, a chemical or an electrical event is not a process. When a content enters into the proximal explanation for why a process happens as it does, that process is said to access that content^5^. Suppose that in order to explain why John draws the conclusion that Steve is mortal from the premise that Steve is a human being, you use a syllogism that relies on the premise that all human beings are mortal. This is a proximal explanation. Therefore, in our terminology, you are assuming that John’s transition from the belief state with the content “Steve is a human being” to the state with the content “Steve is mortal” is caused by John accessing the content “All human beings are mortal.” Now, suppose John formed the belief that all human beings are mortal from reading a book, and he read the book because he believed that books are useful. The belief that books are useful has thus entered into an explanation for why John transitions from the former belief state to the latter, but this explanation is not a proximal explanation. Similarly, there are non-proximal explanations of perceptual processes that involve content that do not imply that those perceptual processes access that content.

The idea behind the generic encapsulation thesis is therefore that all the transitions between contentful states in the module are proximally explained in terms of contents of states within the module or the input that the module receives from earlier units in the perceptual hierarchy. We shall say more about the perceptual hierarchy in the “Neural Case Against Encapsulation” section.

Due to its generality, the generic encapsulation thesis is too easy to satisfy and seems to be theoretically unattractive. For example, one might easily find a small neuronal assembly or a single neuron in the visual system that is informationally encapsulated. However, it is not clear how the truth of such a thesis would relate to the broader theoretical issues that are often linked to the modularity debate. Some of these theoretical issues include questions about the structure and function of the perceptual system, whether perception is a bottom-up or a top-down process, whether observation is theory-neutral, whether perceptual experience has conceptual content, and whether a foundationalist epistemology is tenable^6^. Rather than focusing on the generic encapsulation thesis, we should therefore focus on its specific variants. We can obtain such specific variants by imposing further functional constraints on the module. If these constraints are properly related to the theoretical issues surrounding the modularity debate, the resulting variants of the thesis would be more theoretically interesting.

In his canonical statement of the modularity thesis in *The Modularity of Mind*, Fodor argues that the purpose of the perceptual module is to provide fast analysis of sensory input on the basis of informationally encapsulated, domain specific, innate, and localized informational processes. The representations that result from this analysis are subsequently provided as input to higher cognitive centers and the action system.

The idea that the outputs of the perceptual modules serve as input to higher systems imposes a functional constraint on the notion of the module. Let us call a representation that could be used by higher cognitive centers and the action system without further processing a *pickup ready representation*^7^. In this case, the functional constraint is that the outputs of the perceptual modules should be pickup ready representations. Adding this constraint to the generic encapsulation thesis would give us the following variant:

### Pickup Ready Encapsulation

There is a component of the perceptual system that gives rise to pickup ready representations and is informationally encapsulated.

A module’s functional role in providing input to other systems is not the only theoretical issue that is relevant to the encapsulation thesis. One reason for the earlier surge of philosophical interest in modularity has been whether observation is theory-laden, with some modularists arguing that encapsulation suggests that observation is theory-neutral (see Fodor, 1984; Raftopoulos, 2001). But obviously, not just any form of the encapsulation thesis would have this implication. Suppose, for example, that Pickup Ready Encapsulation is correct and there is a visual module that gives rise to pickup ready representations. Still, it might be the case that the outputs of this alleged module are *pre-observational* in the sense that there is more processing that has to happen over these outputs before they give rise to what can be properly regarded as observation. Since there is still the possibility that these additional processes are not encapsulated, pickup ready encapsulation and the theory-ladenness theses could both be correct at the same time.

The same observation applies to the link between the encapsulation thesis and epistemic issues. Siegel (2012), for example, has argued that some types of encapsulation failure threaten a foundationalist approach in perceptual epistemology. If perception is influenced by background beliefs, then a potentially problematic circularity threatens the idea that perception can serve as the foundation for justifying beliefs. Thus, a foundationalist who accepts Siegel’s arguments might be interested in saving encapsulation. But again, for similar reasons to those that show that it does not disprove the theory-ladenness thesis, Pickup Ready Encapsulation may not serve this purpose.

We therefore need to impose further constraints on Pickup Ready Encapsulation to establish a better link with the above theoretical issues. One possible constraint is to restrict encapsulation to person-level representations. Adding this constraint would give us the following thesis.

### Person-Level Encapsulation

There is a component of the perceptual system that gives rise to person-level pickup ready perceptual states and is informationally encapsulated.

The above version of the thesis gets closer to the theoretical issues surrounding encapsulation. However, those who submit to common forms of internalist epistemology would not find this move very satisfactory. On such views, a state is capable of epistemic evaluation in so far as it belongs to a domain that a subject can access through reflection. Those who accept this form of internalism also have a tendency to demand that a state counts as an observation in so far as it is available to reflection. This suggests stronger constraints on the encapsulation thesis; constraints that would link it to the possibility of reflection. One such constraint is that the outputs of the encapsulated module have to be phenomenally conscious. This is because there is an intuitive connection between phenomenal consciousness and observation. One might even say that it is nomologically necessary that a subject, S, observes an object (or property) only if the subject has a phenomenally conscious representation of the object (or property). In fact, it is not uncommon to characterize the effect of cognition on perception in terms of its effect on the phenomenal content of perceptual experience (for example, see Macpherson, 2012). Adding this constraint will give us a new thesis.

### Phenomenal Encapsulation

There is a component of the perceptual system that gives rise to phenomenally conscious representations and is informationally encapsulated.

However, those who think that there is a gap between phenomenal consciousness and access consciousness might question the link between phenomenal encapsulation and observation (see Block, 1995, 2005). The reason could be that in order for a representation to qualify as an observation, it should be access conscious in the sense that it has to be readily available for verbal report, voluntary control of action, and other personal-level functions^8^. In this sense, however, a phenomenally conscious representation might not be access conscious. We should therefore distinguish Phenomenal Encapsulation from another version of the generic thesis.

### Access Encapsulation

There is a component of the perceptual system that gives rise to access conscious representations and is informationally encapsulated.

So far we have four versions of the encapsulation thesis. These theses are somewhat independent in that one might be false while the others are true. Are there any of these theses that should be regarded as the central encapsulation thesis? We think the answer is negative. A pluralist view according to which there are different versions of the encapsulation thesis that respond to different theoretical demands is, in our view, the correct view. Nevertheless, our focus in this paper will be on Access Encapsulation. This is not because we think perceptual states have to be access conscious. Our main reason is that in comparison to the other versions of the thesis, Access Encapsulation bears the most clear relationship to the broader philosophical issues surrounding the modularity debate. It’s rather intuitive that the failure of Access Encapsulation, even if turns out that the other versions of the theses are true would be philosophically significant. But whether the failure of other forms of encapsulation in those cases where Access Encapsulation remains intact has important philosophical implications or not is less intuitively clear. So our focus in this paper will be on access encapsulation.

The possible constraints that we have discussed so far concern the output of the module. Obviously, output is not the only feature that is relevant for determining the function of a module. One also needs to determine what serves as input to a module. It might seem natural to assume that the input to the perceptual modules should be equated with the physical energy at the level of sensory receptors. On such a view, modules start where sensory receptors start. But this is not the only option. Fodor’s distinction between transducers and input analyzers can help us see why (Fodor, 1983). On Fodor’s view, transducers are in the business of transforming one form of physical energy—for instance, luminance and hue—to another—for example, action potentials in the neuronal axons. According to Fodor, transducers do not perform any computation, and since perceptual modules start where computation starts, modules do not start at the boundaries of sensory organs. The same could be said about any units that transfer and relay the signal from transducers without performing any computations over them. So on a broadly Fodorian view, modules would be higher up in the perceptual hierarchy after units that transduce, transfer, and relay the signal without performing computations.

Fodor’s distinction between the components of the perceptual system that perform computation and those that do not is controversial. Even if we accept it, it is not completely clear why the lower boundaries of the module have to coincide with the boundaries of computation. But the point of mentioning Fodor’s distinction here is not to defend or reject it. The point is that there are at least two possible ways to draw the lower boundaries of a module. One option is to hold that the visual module starts where the boundaries of the sensory organs start. The other option is to hold that the visual module starts further upstream in the visual system. One principled way to determine where it starts would be to point to where computation starts. We thus get two new versions of the encapsulation thesis.

### Primary Encapsulation

There is a component of the perceptual system whose lower boundaries coincide with the sensory receptors and is informationally encapsulated.

### Middle Encapsulation

There is a component of the perceptual system whose lower boundaries are further upstream from transducers, transformers and relay centers, and is informationally encapsulated.

**FIGURE 1:**
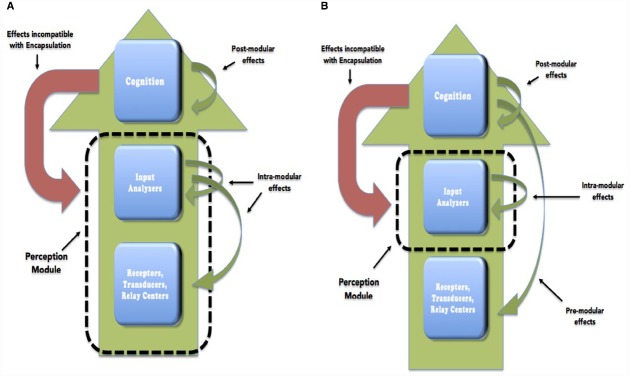
**Primary (A) and middle encapsulation (B) are represented.** We use Fodor’s distinction between central cognition and the various components of the perceptual system. Green arrows in each figure represent flow of information that is compatible with the corresponding version of encapsulation. Red arrows represent flow of information that would be incompatible with encapsulation.

Obviously, the significance and the strength of the encapsulation thesis also depends on whether it is a primary form of encapsulation or a middle form. In this paper we will consider the empirical status of both theses (see Figure 1 for an illustration). Unless explicitly noted otherwise, when we refer to the encapsulation thesis we will mean the disjunction of Primary Access Encapsulation and Middle Access Encapsulation. However, middle encapsulation is the dominant thesis among modularists and we will pay more attention to it. Furthermore our discussion will mainly focus on vision. Accordingly, we will be focusing on whether there is an encapsulated visual module.

One last point before we move on deserves emphasis. The encapsulation thesis is stronger than the oft-discussed thesis that perceptual modules are cognitively impenetrable (see Pylyshyn, 1999; Payne, 2001; Raftopoulos, 2001, 2014; Macpherson, 2012; Stokes, 2013). The main difference here is that informational encapsulation does not only concern cognitive contents. If the cognitive impenetrability thesis is true, then no cognitive state from outside the module can be accessed by processes inside the module. On the other hand, if the encapsulation thesis is true, then no contentful state outside the module, whether it can be regarded as cognitive or not, can be accessed by the processes within the module.

## The Psychological Case Against Encapsulation

Our goal in the following sections is to show that purely psychophysical studies cannot provide sufficient evidence against encapsulation. We do so by identifying three challenges that psychophysical studies must meet. They are the post-perceptual challenge, the intra-modular challenge and the pre-modular challenge. We then argue that no psychophysical study could jointly meet all three of them.

### The Post-Perceptual Challenge

Consider an experiment in which subjects are shown images in which an individual is holding an object that is difficult to identify. Suppose that the results show that whether the subjects would report seeing a gun or a tool depends on the race of the individual holding the object (Payne, 2001). One interpretation of these findings is that implicit racial biases affect the percept, or, in other words, the way the object is seen^9^. Another interpretation is that implicit racial biases do not influence the way the object is seen, but only influence the subject’s post-perceptual judgment about the identity of the object. The difference between these interpretations is relevant to the encapsulation thesis. The first interpretation is potentially incompatible with the encapsulation thesis because it shows that the processes that give rise to a percept can be influenced by factors outside the alleged visual module. However, on the second interpretation, the effect that implicit racial biases have on the subject’s performance is mediated by its effect on post-perceptual judgment. Thus, this interpretation is compatible with the encapsulation thesis. Therefore, in order to show that this finding is potentially troublesome for the encapsulation thesis we need to be able to rule out the post-perceptual interpretation.

The point of this example could be easily generalized. In the above case, the percepts and the behavioral response are mediated by judgment. However, the processes that influence the link between percepts and behavioral responses are not confined to judgment. In principle, it is possible for other mechanisms such as attention, inference, recognition, memory, various forms of response bias, etc., to intervene between the output of a module and a behavioral response. Effects that result from cognitive influences on the processes that link percepts to behavioral response should not count as evidence against the encapsulation thesis. A successful experiment has to be able to determine that the effect does not occur at a post-perceptual level. We therefore call this the post-perceptual challenge.

Meeting the post-perceptual challenge is not easy. Psychophysical studies of effects on perception have to rely on studies of perceptually-guided behavior. But effects on perceptually-guided behavior can happen in two ways: either by affecting the processes that lead to the formation of a percept or by affecting the processes that translate the percept into a behavioral response. Obviously, effects that result from cognitive impact on the latter stage should not count as evidence against encapsulation. There is, therefore, always a possible post-perceptual explanation that needs to be ruled out. Psychophysical experiments must meet this challenge if they are to provide evidence against the encapsulation thesis.

Some theorists have argued that one strategy to meet the post-perceptual challenge is to diminish the number of tasks that are needed to link a percept to a behavioral response. Stokes (2013), for example, argues that in some studies where the stimulus is present during the response phase, it would be implausible to hold that the pattern of response emerges from post-perceptual factors. For example, in Levin and Banaji (2006), subjects are asked to match the degree of luminance of a grayscale patch to the degree of luminance of pictures of faces. These studies show that the presence of labels (or typical race-indicating facial features) influence matching behavior. Faces that are labeled BLACK (or exemplify typical black facial features) are matched to darker patches than faces that are identical in luminance but are labeled WHITE (or show typical white facial features). Stokes argues that it would be implausible to explain away these results as emerging from post-perceptual judgment. The main reasons is that such explanations would have the implication that although subjects have distinct phenomenal experiences of the luminance of the gray scale patch and the picture they match to it, they classify them as having the same luminance. This, in Stoke’s view, renders these interpretations comparatively less plausible.

It is correct that post-perceptual explanations in the above match-to-sample experiments have a lower degree of plausibility in comparison to experiments that rely on memory, but this is clearly insufficient to show that they are implausible explanations *per se*. We should note that there could be phenomenally non-identical experiences that are nonetheless phenomenally indistinguishable. In other words, a subject might have different experiences that she cannot distinguish from each other^10^. If this is plausible, then it is plausible that for any particular experience that a subject has, there is a range of phenomenally non-identical but indistinguishable experiences that the subject could match to this experience in an experimental setting. In other words, as long as two experiences are phenomenally indistinguishable, it is not implausible that a subject would classify them as identical, even when the experiences are non-identical. Perhaps Stokes thinks that post perceptual explanations, in these cases, are implausible because they imply that two phenomenally distinguishable experiences are judged to be identical. However, there is nothing in the aforementioned studies that demonstrates this. Therefore a post-perceptual explanation in these cases is not clearly implausible^11^^,^^12^.

A second possible strategy to rule out post-perceptual explanations is to draw on the resources of signal detection theory (SDT). In fact after the advent of SDT, some psychologists quickly started to use this theory to distinguish perceptual effects from post-perceptual ones^13^. However, we believe that the perceptual vs. post-perceptual distinction that is based on SDT criteria is orthogonal to the perceptual vs. post-perceptual distinction that is operative in the debate between the modularists and the anti-modularists. Let us elaborate on this by first explaining why some have thought that SDT can help us distinguish between perceptual and post-perceptual processes.

Those who apply SDT to perception assume that our response mechanisms have the appropriate built-in structure to distinguish signal from noise. It is further assumed that this feature of response mechanisms can be used to distinguish effects on the response stage from effects on prior stages.

Imagine a task in which subjects are tasked with discriminating between pictures of dogs and non-dogs by pressing a button. Suppose subjects are more prone to classify images as dogs if they hear a story involving dogs before seeing the pictures. How can we figure out whether this is the result of effects on a perceptual detection stage during which a perceptual unit detects the presence of dogs or a post-perceptual response stage during which a response unit responds to the detection stage?

From the standpoint of SDT, informational connections are always noisy. Because of this noise, sometimes the detection unit is not “telling” the response unit that what it sees is a dog, but it might “sound” to the response unit that the signal is “dog.” The basic way that the response unit solves this problem is to adjust a response threshold (or a response bias). When the input exceeds the threshold the response unit will treat it as a signal, and if it is below the threshold it will be treated as noise. The central assumption behind the SDT approach is that when the input to a response unit remains constant, adjusting response thresholds is the only way to affect the behavior of this unit. So, if cognition is making you more prone to classify a picture as a dog by affecting the post-perceptual response stage, it must be doing so by lowering the response threshold to dogs.

This assumption takes us a long way. Whether an effect is a threshold effect in this sense or not can be empirically determined. This is due to an interesting feature of threshold effects. Increasing the threshold reduces the number of cases where noise is falsely treated as signal (fewer non-dogs classified as dogs). However, this has the cost of increasing the number of false negatives, namely, cases where a signal is falsely treated as noise (more dogs classified as non-dogs). Decreasing the threshold, in contrast, decreases false negatives (signal treated as noise) with the cost of an increase in false positives (noise treated as signal). Changes in threshold are therefore essentially accompanied by a coupling between false negatives and false positives. Non-threshold mechanisms, in contrast, need not give rise to a coupling pattern. So one way to find out whether an increase in correct responses to a stimulus type is the result of the adjustment of a threshold is to figure out whether there is a coupling between false negatives and false positives. And in principle, one can detect whether there is such coupling if one has a large data set of responses that can be statistically analyzed^14^. The upshot is that threshold effects can be empirically distinguished from non-threshold effects.

How can this help us distinguish perceptual effects from post-perceptual effects? If post-perceptual effects are threshold effects we can distinguish them from non-threshold effects. But, as was quickly noted, some perceptual effects are also threshold effects. So, finding out that an effect is a threshold effect will not tell us that it is post-perceptual. However, those who think that SDT can help us solve the problem assume that post-perceptual effects are essentially threshold effects. So finding out that an effect is not a non-threshold effect is evidence that it is not post-perceptual^15^.

We can now see the conceptual problem with applying SDT to the task of distinguishing perceptual from post-perceptual effects. The assumption behind this approach is that post-perceptual effects are essentially threshold effects. But it is not clear why we should accept this assumption. There is no conceptual connection between being post-perceptual and being a threshold effect. Of course, some examples of post-perceptual effects, e.g., effects of bias, are plausibly threshold effects. But there is no reason to assume that what is true of these cases generalizes to all post-perceptual cases. Moreover, there is no reason to assume that what is true of the perceptual system does not generalize to the response system. Everyone agrees that the perceptual system can get better at figuring out what happens around us in a way that reduces false positives (or false negatives) without increasing false negatives (or false positives), but that need not involve threshold mechanisms. If this is true, then why should we assume that there could not be non-threshold improvements in how the response system figures out what the perceptual system is “telling” it? After all, there is uniformity at the neural level in that the same basic mechanisms in both the perceptual system and the response system govern the propagation of neural activity. Why should things be different at the psychological level of description?

We thus conclude that it is far more difficult to rule out post-perceptual explanations with psychological methods than opponents of encapsulation usually think. It would be wrong to think that post-perceptual explanations of “online” experiments are implausible. And there is no reason to assume that the distinction between perceptual and post-perceptual mechanisms maps to the distinction between threshold and non-threshold mechanisms. In so far as *d*′ and other parameters are measures of the latter distinction, there is no reason to assume that they can be used to meet the post-perceptual challenge.

This is not to say that post-perceptual explanations could never be ruled out. We think that in non-borderline conditions and in the absence of confounding factors it should be uncontroversial that subjective reports about the perception of a stimulus or its detectability are good indicators of the existence of percepts. After all, it is mainly on the basis of subjective reports that everyone agrees that there is a switch between percepts during binocular rivalry. However, as we shall argue soon, there is an interesting interplay between the different challenges that makes it the case that results that are hard to explain post-perceptually are more susceptible to the pre-perceptual or intra-modular challenges^16^.

### The Intra-Modular Challenge

**FIGURE 2:**
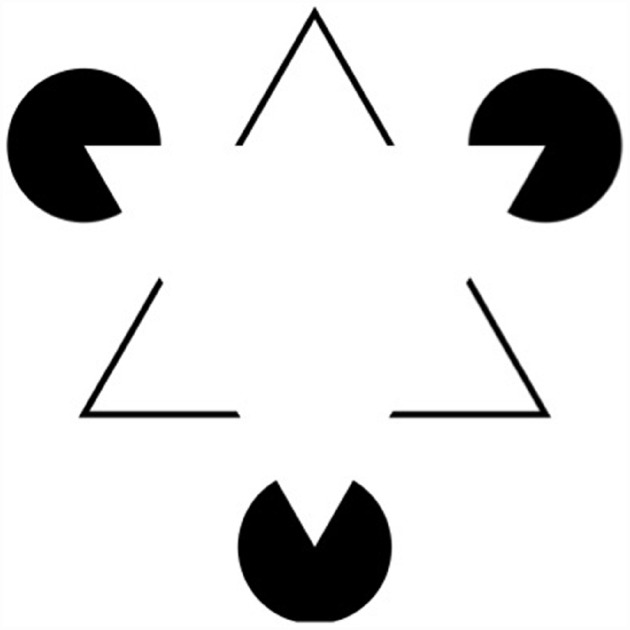
**The perception of these figures, known as Kanizsa triangles (or squares) can be explained by interactions within the visual module**.

As we saw in the previous section, one requirement a study must meet in order to provide evidence against encapsulation is to show that the locus of an effect is not post-perceptual. But this is not sufficient to refute the encapsulation thesis because it is possible that the origin of the effect is *intra-modular*. If both the locus and the origin of an effect are within the visual module, then the effect cannot count against encapsulation. In fact, putting both the locus and the origin of an effect inside the module is a common mode of explanation of what are called contextual effects. Contextual effects occur when the perception of individual elements within a visual scene are influenced by other elements within the scene. A famous example of this is the phenomenon of amodal completion where we perceive elements in the visual scene for which no direct information in the proximal stimulus is present (see Figure 2 for an illustration of this phenomena).

The common explanation for contextual effects is that although stimuli in different areas of the visual field are processed by different and partially independent units in the visual module, these units can sometimes interact with each other through intra-modular connections. These connections embody knowledge. But this knowledge is embedded within the module, and the fact that it can influence the output of the module is compatible with the encapsulation thesis.

This observation generalizes beyond contextual effects. Some alleged effects on perception can be explained as intra-modular effects. Therefore, a second challenge for an empirical study that aims to provide evidence against the encapsulation thesis is to rule out intra-modular interpretations of the findings. We call this the *intra-modular challenge*.

**FIGURE 3:**
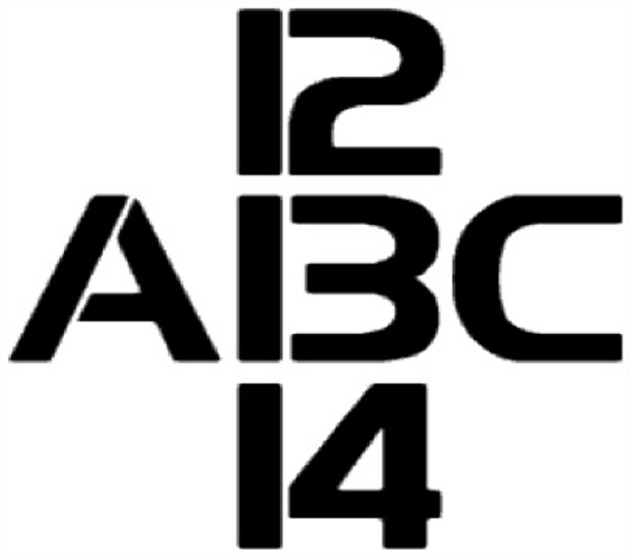
**In this figure, the object in the middle can be perceived either as the number 13 or the letter B.** It’s possible to explain this effect via interactions within the visual module.

Whether a study can meet this challenge partly depends on how we draw the boundaries of the visual module. Consider the experiment at the beginning of the previous section where the way that subjects categorized an ambiguous image was influenced by the race of the individual holding the object. It might seem natural to assume that categorizing an object as a gun or a tool, or categorizing individuals as belonging to different races, is a post-modular matter. But it has not been definitively established that the visual module cannot represent object categories or race categories. Consider Figure 3 in which we can perceive the shape in the middle either as number 13 or as letter B. In our view, it is not implausible at all that the difference between perceiving the letter as a B or as number 13 is a perceptual matter. If so, then the visual system might be able to represent categories including object categories and racial categories^17^. And if this is the case, effects of racial categories on how objects are categorized can be potentially explained away as intra-modular effects.

The intra-modular challenge becomes more serious if we allow, as modularists like Pylyshyn do, that the boundaries of modules are flexible and can change as a result of perceptual learning. For example, on a view like this acquiring expertise in reading written text partly consists in the automatization and encapsulation of the processes that give rise to representations of the semantic properties of a word. These processes thus become part of the visual module. So acquiring fluency in reading written text partly consists in the fact that the visual module now comes to represent semantic categories and the association between these categories and orthographic markers^18^. As such, an alleged effect of word meaning on visual experience of words can result from an intra-modular effect.

There is no reason to think that such an account could not be generalized beyond semantic categories. In principle, as a result of learning, many complex properties and their association with simpler visual markers such as colors and shapes can come to be represented by the visual module. If so, the effects of the representation of these properties on vision can potentially be explained away as intra-modular.

To see the significance of treating perceptual learning as a form “modularization,” consider the alleged effect of race indicative facial features on color perception (Levin and Banaji, 2006). Participants in the debate normally assume that representation of race is a post-perceptual matter. If so, the origin of such effects would be outside perception, and as a result, the discussion of these effects has mostly focused on whether they can be ruled out post-perceptually. But if we allow that the boundaries of the visual module could expand with learning, the intra-modular explanation would also become an option. Perceptual learning might result in the modularization of both the representations of facial features and their association with a specific color. This would also explain why such effects are resistant to explicit beliefs to the contrary and are usually classified as effects of implicit beliefs. One could therefore explain away the effect of facial categorization on color perception as an intra-modular effect.

The idea that perceptual learning can result in the representation of new complex properties might seem incompatible with modularism. It seems plausible to assume that new complex properties come to be represented by the visual module in so far as at some point during the learning process there have been influences from outside the visual module which directed the learning process. However, one can distinguish between two types of learning, namely, Additive Learning and Revisionary Learning. Revisionary learning changes the parameters of existing processing capacities within the module. Revisionary learning, when it happens as a result of access to information outside the module is incompatible with our definition of encapsulation. Additive Learning, in contrast, occurs as a result of the addition of new processing capacities to the visual module, e.g., new capacities which often allow for the representation of new properties. This type of learning does not conflict with the encapsulation thesis as we define it^19^.

These observations should show that meeting the intra-modular challenge is not as easy as it initially might seem. Now let us consider a third challenge for anti-modularists.

### The Pre-Modular Challenge

We have so far argued that anti-encapsulationist studies have to face the challenge of ruling out post-perceptual and intra-modular explanations of their findings. However, ruling out these explanations is not sufficient for refuting the encapsulation thesis. Suppose, for example, that it has been empirically demonstrated that expert bird-watchers are faster and more accurate in visually recognizing birds than non-experts. Let us also suppose that we have successfully ruled out post-perceptual and intra-modular explanations for this finding. Still, there is the possibility that the main cause of this difference in performance lies in the fact that experts employ more efficient visual search strategies. In short, experts know where to look. As a result, when an expert and a non-expert look at the same bird, the input that the visual module of the expert typically receives is different from the input that the visual module of the non-expert receives. It is therefore possible to explain the effect of expertise in terms of pre-perceptual differences in input. This illustrates the *pre-modular challenge*.

The pre-modular challenge is not confined to effects of expertise, and can in principle be employed to explain away many allegedly cognitive effects on perception. For example, some priming effects on perception can be explained as pre-modular effects. Thus a third challenge for an anti-encapsulationist empirical study is to rule out pre-modular interpretations of the findings. We call this the pre-modular challenge.

Note that the breadth of the pre-modular challenge partly depends on whether we accept the primary or the middle version of the encapsulation thesis. As we noted in the “Encapsulation Thesis” section, according to Middle Encapsulation the visual module starts somewhere in the middle of the visual hierarchy. Specifically, it does not start where the retina starts. On such a view, some attentional shifts could change the inputs that a module receives by modulating the activity of the transport or relay units prior to the visual module (see O’Connor et al., 2002; Cudeiro and Sillito, 2006; McAlonan et al., 2006). Such effects would be thus compatible with middle encapsulation.

To meet the pre-modular challenge posed by Middle Encapsulation, one needs to rule out that the observed effects are effects of pre-modular attention. Primary Encapsulation, in contrast, is incompatible with attentional effects on relay centers. In order to meet the pre-modular challenge posed by Primary Encapsulation, one only needs to rule out that the observed effects result from visual search strategies (for example, changes in direction of gaze and saccadic movements). The pre-modular challenge is therefore harder to meet if our aim is provide evidence that Middle Encapsulation fails, as opposed to the Primary Encapsulation. This is so because, in addition to visual search strategies, attention may affect the relay and transport centers between the retina and the lower boundaries of the visual module.

The effects that result from where a subject looks could in principle be ruled out by controlling for factors such as eye movements (whether saccadic or deliberate), but there are other types of attention that one must rule out. Although the most frequently cited such attentional effect result from spatial attention, it is not uncommon for modularists to also appeal to feature-attention. Pre-modular feature attention occurs when a subject’s attention to a specific feature changes the activity of units that relay activity pertaining to that feature.

How could we rule out attentional effects, of any type, by purely psychophysical experiments? One thought here might be that attentional effects are weak and are confined to spatial properties and simple features. So one way to meet the attentional challenge is to find robust and complex effects on perception.

However, it is not clear that the effects of attention are always weak and simple. It might be true that pre-modular attentional shifts can only cause minor changes in the input that a module receives. However, minor changes in input can result in Gestalt-like switches in the way that the input is processed. Consider, for example, Figure 3 again, in which the ambiguous figure can be interpreted both as number 13 or as the letter B. As we noted, it is possible to interpret this effect as post-perceptual or intra-modular. But a third option is that the difference between the two cases emerges from differences in pre-modular attention. For example, perception of the shape as a 13 could result from paying more attention to the gap between the curved and the horizontal lines, and perceiving it as a B could result from moving attention away from the gap. Our visual module is therefore receiving two different patterns of input in the two cases. And although this difference might be minor, it may be sufficient to cause a Gestalt shift in the way that the input is processed by the visual module. Accordingly, a minor attentional change might result in a robust difference in whether the perceptual system classifies the input as a B or a 13^20^.

It is indeed hard to see how one might be able to meet the pre-modular challenge by purely psychophysical methods. One might for example try to design experiments in which the attentional difference between experimental and control groups or within subjects during different experiment conditions are minimized. However, we are aware of no such studies. We therefore think that meeting the pre-modular challenge is especially difficult if our goal is to refute the middle encapsulation thesis.

### The Interplay Between the Challenges

As we noted earlier, there is also an interesting interplay between the above three challenges in that attempting to meet one of them often makes meeting the others more difficult. One might, for example, argue that the early occurrence of an effect is a reason to rule out that it is a post-perceptual effect. However, this would obviously make ruling out intra-modular or pre-modular explanations more difficult.

Another example involves appealing to cognitive manipulability in order to rule out post-perceptual explanations. Consider the implicit bias studies that show that subjects who are otherwise completely unaware of having racial biases are more prone to report a blurry image as a gun when it’s held by an African American individual (Payne, 2001). Discussion of this effect has often focused on explaining it away as a post-perceptual^21^. But now suppose that in an attempt to rule out the post-perceptual explanation, we show that the effect cannot be manipulated by, say, informing the subjects of their bias. In other words, the effect turns out to be resistant to cognitive manipulation. This would give us some reason to think that the effect is not post-perceptual. The problem is that this would also increase the likelihood that the effect is intra-modular. In general, ruling out the post-perceptual explanation of an effect by showing that it is resistant to cognitive manipulation increases the likelihood that the effect is intra-modular. So, here too our attempt to meet the post-perceptual challenge makes it less likely that we can meet the intra-modular challenge.

We have considered three challenges that psychophysical studies must meet in order to provide evidence against the encapsulation thesis. We have argued that meeting these challenges individually, and in conjunction with each other, is much more difficult than what has been often assumed. We conclude that it is unlikely that one would be able to rule out the encapsulation thesis with purely psychophysical studies.

In a forthcoming BBS target article on this issue, Firestone and Scholl take a more radical line, arguing that almost all psychophysical findings in support of top-down influence has been debunked. Since most psychological models of perceptual processing are purely bottom up, they conclude that empirical evidence favors the encapsulation thesis.

We do not think that the claim that all psychological evidence against encapsulation has been debunked is correct. In our view, the evidence is inconclusive. But one who accepts this might still think that rather than concluding that psychophysics cannot settle the debate, the correct conclusion should be similar to Firestone and Scholl’s line that is, we should embrace the encapsulation thesis.

This line of thought assumes that the encapsulation thesis is the default and has to be upheld unless there is psychophysical evidence against it. But we do not see any empirical reasons to accept the encapsulation thesis as default. It is true that many working models of psychological processes are bottom up. But that is mainly because many modelers assume the bottom-up model as an *a priori* meta-constraint on psychological theorizing. We think that given the newly emerging predictive coding models of perceptual phenomena, this pattern will gradually change (see Clark, 2013; Hohwy, 2013, for references).

Some might also think that the upshot of this conclusion should be skepticism about the empirical resolution of the debate between modularists and anti-modularists. However, we think that the proper reaction is to combine psychophysical studies with other empirical sources of evidence. In the next section we focus on one of these sources, namely, the evidence from neuroscience. As we shall argue, considering this sort of evidence, tilts the balance of the empirical evidence in favor of the anti-modularist.

## The Neural Case Against Encapsulation

As we saw in the previous sections, the psychological evidence against the encapsulation thesis is at best inconclusive. In the following sections, we examine the plausibility of the encapsulation thesis from the perspective of neuroscience. This goes against the common approach in the recent literature that does not engage with this body of evidence. Our focus will be on the access version of the encapsulation thesis. As a reminder, this is the thesis that there is a component of the visual system that gives rise to access conscious representations and is informationally encapsulated. As before, we will simply refer to this thesis as the encapsulation thesis. We argue that recent findings about the connectivity structure and activity dynamics of the visual system militate against this thesis.

Our guiding question is whether a neural correlate of an encapsulated module could be identified in the human visual system. We consider two strategies for demarcating this alleged neural correlate. After a quick introduction to the structure of the visual system, we consider identifying the perceptual module with a neuroanatomically demarcated area of the visual system^22^. We argue that although an area of the cortex that would correspond to the functional profile of a module could be roughly demarcated, empirical results show that this area is not functionally encapsulated. We then consider a strategy that identifies the neural correlate of the visual module with a temporally identified process that happens in a roughly demarcated neuroanatomical region of the cortex. We argue that although an encapsulated visual process could be identified, empirical results suggest that this process fails to give rise to access conscious representations. Lastly, we anticipate a few replies to our argument and respond to them.

### The Neuroanatomical Strategy

**FIGURE 4:**
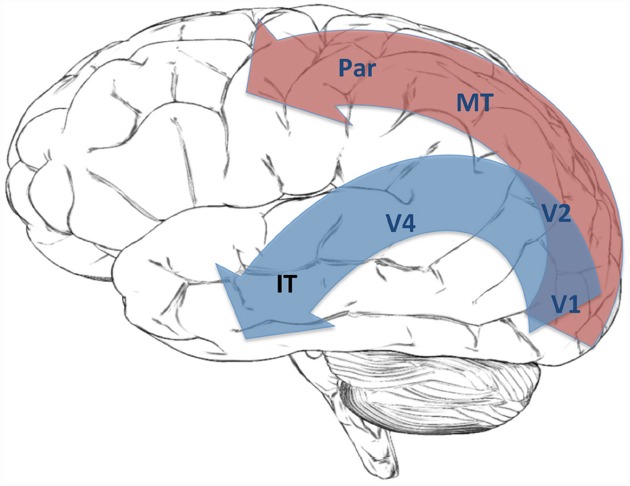
**This figure shows the Dorsal Stream (red) and Ventral Stream (blue) in the visual hierarchy**.

Neuroscientific orthodoxy regards the visual system as a hierarchical structure. Activity starts at the retinal receptors and passes through the retinal ganglion cells to the lateral geniculate nucleus (LGN) in the thalamus. This is an area within the brain stem that is often regarded as a relay center for sending information to cortical areas. In the case of vision, projections from LGN connect it to the primary visual cortex (V1), after which the visual pathway divides into ventral and dorsal streams. The ventral stream includes areas V2, V4 and the temporal lobe. The dorsal stream includes areas V3, MT and the parietal cortex^23^. The visual system thus manifests a fork-shaped hierarchical structure (Figure 4).

This neuroanatomical hierarchy also corresponds to physiologically and functionally specified hierarchies. Physiological studies show that neurons in the visual cortex could be ordered with respect to the size of their receptive fields, that is, the area of the retina that a neuron responds to. Interestingly, the ordering on the basis of receptive field sizes roughly corresponds to the position of a neuron in the neuroanatomical hierarchy; the higher the neuron in the hierarchy, the larger its receptive field. According to orthodoxy, the neuroanatomical hierarchy also roughly corresponds to a functional hierarchy. Different neurons respond to the presence of different types of stimuli in their receptive fields. For example, some neurons respond to the presence of edges, some respond to motion, some respond to colors, and some respond to complex shapes. This is often called the tuning function of a neuron. It is commonly held that tuning functions can also be ordered with respect to their complexity. For example, detecting a shape is more complex than detecting an edge. This hierarchy of functional complexity also roughly corresponds to the neuroanatomical hierarchy: neurons higher on the neuroanatomical hierarchy have more complex tuning functions.

If the boundaries of the visual module are neuroanatomical they should naturally fall somewhere within the visual hierarchy. The question is where. We shall start with the minimal working hypothesis that the visual module starts in area V1 and extends to V4 in the ventral stream. We shall call this area the lower visual system (LVS).

A few points about identifying the alleged visual module with LVS are in order. First, LVS does not include areas earlier than V1 in the visual hierarchy such as retinal receptors, ganglion cells, and LGN. The encapsulation of LVS would, therefore, correspond to the Middle Encapsulation thesis as characterized in the “Encapsulation Thesis” section. Second, we have not included the dorsal stream in LVS. The initial rationale for this is that our focus here is on Access Encapsulation and it is common to assume that the dorsal stream does not give rise to access conscious representations^24^. Third, we have not included areas higher than V4 in LVS. The main rationale for these limitations is to simplify the structure of the discussion. After considering whether LVS is encapsulated, and arguing that it is not, we consider modifying the minimal working hypothesis by adding areas earlier than V1, the dorsal stream and areas higher than V4. The basic question to consider at this stage is whether LVS is encapsulated. We think it is not. What follows is a review of some of the main findings that support this claim.

Recent research shows that the receptive field sizes of neurons, even in the V1 area, change over time (Gilbert and Wiesel, 1989; Hupe et al., 2001; Li and Gilbert, 2002; Stettler et al., 2002; Bair et al., 2003; Angelucci and Bressloff, 2006; Gilbert and Li, 2012; for a review, see Gilbert and Li, 2013). Whereas a neuron’s early response to stimuli (<100 ms) reflects the presence of a stimulus in its classical receptive field, a neuron’s later activity (after 100 ms) is sensitive to the presence of flanking stimuli outside its receptive field. These effects are often referred to as contextual effects. The existence of contextual effects is relevant to encapsulation because V1 neurons do not receive direct input from the areas of retina that fall outside their classical receptive field. Therefore, if a neuron is sensitive to the presence of stimuli outside its classical receptive field, it must receive input from areas other than areas earlier in the visual hierarchy. And if these areas are outside LVS, then LVS is not encapsulated. However, modularists often hold that these contextual effects can be explained in terms of communication between neurons at the same level of neuroanatomical hierarchy (horizontal connections) or recurrent feedback from neurons higher in the visual hierarchy but still within the boundaries of the visual module. So it might be possible to explain away contextual effects in terms of connections within LVS.

However, the exact circuitry underlying contextual effects is still a matter of controversy. There are at least two camps. The first camp holds that horizontal connections are the primary carriers of contextual effects. The second camp holds that the primary carriers of contextual effects are recurrent feedback connections from areas higher than V1, including MT, which is outside LVS^25^. So, whether contextual effects present a threat to the encapsulation of LVS is still a live issue^26^.

A second set of findings that poses a more serious threat for the encapsulation of LVS comes from studies of the circuitry underlying attentional effects on the visual system. These effects are often classified into spatial, feature-based, and object-oriented attentional effects. There are interesting conceptual questions surrounding these distinctions, but for our purpose what matters is the following:

(a) It has been shown that spatial attention modulates neuronal responses in V1, V2, and V4 (Mountcastle et al., 1987; Motter, 1993; Gandhi et al., 1999; Ito and Gilbert, 1999; McAdams and Maunsell, 1999; Reynolds et al., 1999; Crist et al., 2001).
(b) Feature-based attention and task-related effects that are sometimes regarded as attentional effects modulate the activity of V4 neurons (Motter, 1994; Chelazzi et al., 2001; Reynolds and Desimone, 2003; Li et al., 2004; Zhou and Desimone, 2011; Gilbert and Li, 2013).
(c) Object-based attention can modulate activity in areas as low as V1 (Jolicoeur et al., 1986; Roelfsema et al., 1998; Scholte et al., 1999; Lamme and Roelfsema, 2000; Roelfsema et al., 2000).
(d) TMS studies have shown task-specific modulatory effects of expectations in V4 neurons (Morishima et al., 2009).

These findings show that the activity of neurons in LVS modulate with tasks, expectations and attention. The attentional effects are endogenous that is, attentional effects that are not induced by stimuli (exogenous attention). So the origin of these effects lies in areas higher than LVS. Moreover, these effects are content-sensitive. It is as though the neurons inside LVS know what task a subject is performing or which aspect of the stimulus the subject is attending to. This implies that LVS is not informationally encapsulated.

We have described three sets of findings that challenge the encapsulation of LVS. To summarize, (a) there are well-established contextual effects on LVS and it is still a matter of controversy whether these effects could all be explained in terms of connections between neuronal assemblies within LVS, (b) there are well-established effects of spatial attention, feature attention, and object attention effects on LVS that originate from areas outside LVS, and (c) there are well-established task related and expectation related effects on LVS. We therefore think that LVS is not informationally encapsulated and this puts pressure on the encapsulation thesis.

It might be argued that attentional effects are not incompatible with encapsulation. Later in the paper we will argue that this response fails, but we shall discuss a more pressing question first. Could the challenge for the encapsulation thesis be simply removed by identifying the visual module with a neuroanatomical area that is different from LVS? We think the answer is negative. Let us elaborate.

One could modify the thesis that the visual module is identical with LVS by either adding areas to it, subtracting areas from it, or by a combination of these two strategies. We do not think that any of these modifications would help the modularist. For example, consider extending the alleged visual module by adding area MT to LVS. This might seem to help the modularist because under this modification the feedback connections from MT to lower areas like V4 and V2 would now count as intra-modular effects. But this move has an important cost. Now that area MT is part of the visual module, those feedback connections from higher areas that modulate the activities of MT neurons would threaten the encapsulation thesis. There is ample evidence that there are such feedback connections to MT (Treue and Maunsell, 1996; Treue and Trujillo, 1999; Ninomiya et al., 2012). Now consider the reverse strategy of shrinking the alleged module by, say, subtracting area V4 from the LVS. The benefit of this would be that modulating feedback connections to V4 would now count as post-modular. But the cost is that the well-established effects of V4 on lower areas that were originally classified as intra-modular effects would now be incompatible with the encapsulation thesis.

This problem seems to generalize to any proposal for expanding or shrinking the alleged module by adding an area to, or removing an area from, the upper boundary of LVS. Expanding the boundaries of LVS to include areas higher in the visual hierarchy might accommodate some of the aforementioned effects as intra-modular. But this risks threatening the encapsulation thesis because the higher an area in the visual hierarchy the more it is likely that it receives input from areas further up. This is due to the fact that the hierarchical organization of the cortex gradually fades away as we move up the visual hierarchy and gives way to a non-directional connectivity pattern. Subtracting areas, on the other hand, suffers from the same problem that we described above. It also risks conflicting with the requirement that the outputs of a module should be access conscious.

For similar reasons, it is hard to see how the other ways of expanding or shrinking the visual module, such as adding the dorsal stream, adding areas prior to V1 or subtracting areas from the lower boundary of the module, would help the modularist. We therefore conclude that there is no clear neuroanatomical strategy for demarcating an area in the visual system that is encapsulated and whose outputs are access conscious representations. There is no neuroanatomically identifiable visual module.

### The Temporal Strategy

Neuroanatomical strategies do not exhaust the options for the modularists. One alternative is to partially characterize the neural correlates of the visual module in a temporal fashion. The core insight behind this strategy emerges from a deep and interesting debate over the proper way to establish a mapping between the functional and structural description of the brain. This debate is independent from the debate over modularity, but it would help to say a few words about it first. For a long time, a very influential line of thought among neuroscientists has been that there is a one to one mapping between the fine grained structure of the cortex and its functional description, especially in areas corresponding to the perceptual system. Accordingly, one could say that some V1 neurons have the single function of responding to changes in orientation in a specific area of the visual field. This is what we earlier referred to as the tuning function of a neuron or a neuronal assembly. This idea has been lately challenged by neuroscientists who argue that neurons can perform different functions at different times (see Lamme and Roelfsema, 2000, for a review). A neuron’s tuning function and receptive field sizes can both change as a result of receiving input through recurrent feedback connections. For example, a V1 neuron that responds to orientation in a small area of the visual field in the first 100 ms following the presentation of the stimulus, shows sensitivity to more global and complex features after 100 ms.

Lamme and Roelfsema (2000) expand on this idea by distinguishing between two phases of activity in the early parts of the visual cortex. The first phase, the feedforward sweep, happens in the 40–100 ms window after stimulus onset. The ensemble of neurons that participates in feed forward sweep and their activation pattern is primarily determined by feed forward connections. This is simply because there has not been enough time for recurrent connections to exert their influence on these neuronal assemblies. After 100 ms, horizontal and feedback connections start modulating the activity of the neurons that participated in feed forward sweep and change their tuning functions and receptive fields.

This view, if correct, would have deep and important implications for a wide variety of issues, including the nature of attention and the neural correlates of consciousness. But for our purposes here, the point is that the view provides an attractive alternative for the modularist because it seems capable of dealing with the complications that we raised in the “Neuroanatomical Strategy” section. On this alternative, rather than identifying the neural correlates of the visual module with a specific area of the visual cortex, we identify it with a process that takes place in a neuroanatomical area during a specific time interval. For example, one option is to identify the visual module with the feedforward sweep that takes place in LVS. This strategy seems initially promising because it guarantees the encapsulation of the visual module. Since during the feedforward sweep the activity of LVS neurons is solely determine by feedforward connections, and the neural correlate of the visual module is identified with the feedforward sweep that happens in LVS, then the visual module is encapsulated.

We can now see why some modularists, such as Raftopoulos (2009, 2014), have found the temporal strategy attractive. Raftopoulos does not identify the visual module with feedforward sweep. Rather, drawing on Lamme (2003), he divides the wave of activity after the feedforward sweep into two phases. The first phase is a local recurrent phase that culminates at 120–150 ms after stimulus onset. The second phase is a global recurrent phase that starts around 150–200 ms after stimulus onset and allows for feedback connections from higher cognitive areas. On Raftopoulos’ view, the visual module (what he calls early vision) should be identified with the processes that happen in the lower areas of the visual hierarchy during the first 150 ms and include the feedforward sweep and combine the feedforward sweep with the local recurrent phase.

Despite its initial attraction, however, the temporal strategy could not help the modularist save the encapsulation thesis. Recall that our target is Access Encapsulation according to which there is an informationally encapsulated component of the visual system that gives rise to access conscious representations. But there is ample evidence that the activity in the first 150 ms after stimulus onset is not sufficient for access consciousness (Bridgeman, 1975, 1988; Kovács et al., 1995; Vogel et al., 1998; Rolls et al., 1999; Dehaene et al., 2001; Lamme et al., 2002; Sergent and Dehaene, 2004; Koivisto et al., 2006, 2009; van Aalderen-Smeets et al., 2006; Del Cul et al., 2007; Fahrenfort et al., 2007; Melloni et al., 2007; Lamy et al., 2009; Railo and Koivisto, 2009). On the dominant view, access consciousness requires the availability of representations in a global workspace which does not happen before 300 ms after stimulus onset (see Dehaene and Changeux, 2011, for a review of the relevant literature). What is controversial is whether the earlier phase of activity is sufficient for phenomenal consciousness, which is independent from issues regarding access consciousness. The temporal strategy thus fails to save Access Encapsulation for the simple reason that the activity during the early phase after stimulus onset is not sufficient for access consciousness.

### Do Attentional Effects Entail Failure of Encapsulation?

We have so far argued that given the status of recent neuroscientific findings, it is very unlikely that a neural correlate for a visual module that is informationally encapsulated, and gives rise to access conscious representations, could be identified. Our strategy has, in effect, presented the modularist with a dilemma. The first horn is to identify the visual module with a neuro-anatomically demarcated area of the visual cortex. If the modularist chooses this option, she has to face the challenge of accounting for the existence of feedback connections that modulate the activity of the neurons in early visual areas. The second horn is to identify the visual module with processes that are partially characterized temporally. But choosing this option conflicts with the requirement that the visual module should give rise to access conscious representations. Before ending the paper we want to return to a question that we brought up earlier in the “Neuroanatomical Strategy” section, namely, whether attentional effects are compatible with encapsulation. We consider three reasons for thinking that they are and argue that none of them withstand scrutiny.

We noted, in the “Pre-Modular Challenge” section, that proponents of modularity commonly hold that some attentional effects on perception are mediated by changes in the input to the module from earlier areas and are therefore compatible with the encapsulation thesis. We agreed with the modularists that pre-modular attentional effects do not count as failures of encapsulation. But it might be argued that a similar strategy could be employed against our arguments here. Accordingly, one might argue that the attention-induced modulations of the activities of the LVS neurons are mediated by effects on the units earlier than LVS. Attention-induced modulations of LVS neurons would therefore be mediated by modulations of the inputs to LVS and can thus be explained away as pre-modular.

Three problems threaten this response. First, the aforementioned studies do not mention modulations of pre-modular centers that accompany the attentional effects on LVS. Second, there is reason to think that some of the attention induced modulations in areas such as V4 and V2 could not have mediated by effects on units prior to LVS. This is because some earlier studies of attention induced modulations of areas V4, V2, and V1 could not find any modulation of V1 neurons. In fact, it was only very recently that modulations of V1 neurons have been detected (Luck et al., 1997; Gandhi et al., 1999; Ito and Gilbert, 1999; Maunsell and Cook, 2002)^27^. Any impact of the units prior to V1 on LVS has to be mediated by V1. Therefore the absence of V1 modulation implies that the effects on higher areas, such as V4 and V2, could not have originated from direct effects on earlier units.

The third problem is that it is not clear how the pre-modular strategy could be applied to modulations induced by cases of feature and object attention. Attention induced boosting of the signal corresponding to a specific feature or object requires boosting the activity of a neuronal assembly that represents that feature or object. But it is not clear how pre-modular transducer and relay units could represent objects, or any features except for those for which there are transducers. In fact, within a broadly Fodorian framework that most modularists endorse, representations of objects and representations of most features are post-computational. Therefore, pre-modular units, which are pre-computational, could not represent features or objects. It is therefore unclear how attention induced boosting of the activities of pre-modular units could account for the effects of object and feature attention on LVS neurons.

Attentional effects have sometimes been regarded as compatible with encapsulation because they constitute a state of “readiness.”^28^. However, it is not clear why this should render these effects compatible with encapsulation. It is true that some attentional effects on visual areas happen before the area receives stimulus-induced activities. Such effects would be in some sense pre-perceptual, so “readiness” might be an apt label here. However, it is not clear why the fact that an effect happens prior to stimulus-induced activity makes it compatible with encapsulation. If the higher areas of the cortex could “tell” a V1 neuron that what it is about to “witness” on the left side of the visual field is important and thereby affect the way it processes the input, then the V1 neuron has access to the information in the higher areas. It does not matter whether this access happens prior or posterior to stimulus-induced activity.

A second thing that the “readiness” label might mean is that these effects are not content-sensitive. Suppose there is a perception-booster potion that boosts the readiness and thereby the post-stimulus response of all the V1 neurons to all different types of stimuli in their receptive fields. Then we agree that there is a sense in which this boosting does not qualify as a failure of encapsulation because explaining it does not require appealing to contents. This is because explaining the effect of this potion would not require attributing to the V1 neurons access to any sort of content, e.g., what is the task at hand, what is salient for the task, what should the perceiver attend to, etc. But all the attention induced effects that we have cited here, whether they qualify as cases of “readiness” or not, are content-sensitive effects.

These attentional effects cannot be explained away by saying that they are mediated by pre-modular changes in input. Nor does the fact that some of them happen before stimulus-induced activity render them compatible with encapsulation, since they are content-sensitive effects. We conclude that these attentional effects are incompatible with encapsulation.

## Conclusion

A core thesis in the debate between modularists and their opponents is the encapsulation thesis according to which there is a component of the visual system that is informationally encapsulated from impact from areas higher in the perceptual hierarchy. The secondary literature on the encapsulation thesis has mainly focused on the implications of purely psychophysical findings for this thesis. In this paper, we have pushed against this common tendency in two ways. We have argued that due to methodological limitations, purely psychophysical studies are incapable of resolving the debate between the modularists and their opponents. This gives us some reason to look for other sources of evidence. We have also taken the first steps in this direction by arguing that findings in the past few decades about the neural structure and connectivity pattern of the visual system undermines the encapsulation thesis. We hope that our arguments here help move the debate to the direction of taking the neural data more seriously.

Throughout the discussion, we have also distinguished between different versions of the encapsulation thesis and analyzed its relation to the broader context of theoretical disputes surrounding the modularity debate. These distinctions helped structure and clarify the discussion that followed, and we hope that they will be of service to the continuing debate on the topic.

### Conflict of Interest Statement

The authors declare that the research was conducted in the absence of any commercial or financial relationships that could be construed as a potential conflict of interest.

## Footnotes

1 This is not to say that this property exhausts or is definitional of what a module is. Fodor (1983), for example, attributes nine properties to modules one of which is informational encapsulation.

2 We hold that any information that a process receives is, by definition, input to the process. So we think that defining informational encapsulation in terms of having access only to inputs makes the thesis trivially true and thus non-substantive.

3 An exception to this is Raftopoulos (2009, 2014) who engages with the evidence coming from neuroscience.

4 See Pylyshyn (1999), Durgin et al. (2009, 2012), Raftopoulos (2009), Zeimbekis (2013), and Firestone and Scholl (2014, 2015a,b) for examples of modularist replies. For a review of some of the empirical exchange between modularists and anti-modularists see Witt et al. (2015).

5 Note that this is different from saying that the content enters the explanation of why the process is the process that it is.

6 For some discussion of the theoretical issues concerning the modularity debate (see Fodor, 1984; Churchland, 1988; Fodor and Pylyshyn, 1988; Raftopoulos, 2001; Siegel, 2012; Stokes, 2013).

7 It is not completely obvious what could serve this function. But there are some proposals on the table. For example, on Pylyshyn’s view, modules have to give rise to what he calls “proto-objects” which are themselves the products of two sets of processes within the early visual system: those that compute features such as color, depth, luminance, and motion (Pylyshyn, 1999, p. 361–362), and those that bind these features into a single proto-object by top-down and horizontal processes within early vision. The binding of the features into a proto-object creates representations that are stable and robust enough to be compared with representations in long-term memory for use in recognition and identification (Pylyshyn, 2001, p. 145).

8 Note that a representation could be pickup ready without being access conscious. For example, a representation might be ready to be picked up by systems that are responsible for involuntary action without being access conscious. If, as Milner and Goodale (1995) have argued, the representations in the dorsal stream could control involuntary action without being conscious, the dorsal stream representations are pickup ready but not access conscious.

9 By a percept we mean an access conscious representation in the sense identified in the “Encapsulation Thesis” section.

10 Disjunctivists are well-known for distinguishing between phenomenal distinctness and indistinguishability. But one does not have to be a disjunctivist in order to distinguish between phenomenal identity and phenomenal indistinguishability. One might independently be wedded to the view that phenomenal character is a matter of what belongs to phenomenal consciousness but indistinguishability is a matter of what a subject can distinguish under reflection on phenomenally consciousness contents. So the concepts of phenomenal identity and phenomenal indistinguishability seem to be distinct concepts. The distinction is also motivated by empirical cases such as change blindness that seem paradoxical under a view that would equate phenomenal identity with phenomenal indistinguishability. For, these cases seem to imply that phenomenal indistinguishability is not a transitive relation. Identity, however is a transitive relation. So phenomenal identity and phenomenal indistinguishability cannot be co-extensive.

11 Zeimbekis (2013) employs the same strategy in support of a post-perceptual interpretation of the results of color-matching experiments.

12 Firestone and Scholl (2015a) challenge the Levin and Banaji (2006) experiments by showing that the effects disappear when we blur out race. They therefore conclude that the effects are pre-perceptual. Our goal here has been to show that the post-perceptual explanation would still available even if Firestone and Scholl’s critique of these findings fails.

13 For a review of some of these studies see Pylyshyn (1999).

14 The two crucial parameters in SDT that were assumed to lend themselves to this task are *d*′ (or the sensitivity parameter) and β (or the response bias parameter). The former is the distance between the means of noise and signal-plus-noise distributions. Changes in *d*′ have been taken to indicate changes in the percept and changes in β as indicators of response bias.

15 This is why changes in *d*′ are regarded as indicative of changes in the percept.

16 Wilbertz et al. (2014) and Marx and Einhauser (2015) are example of studies that demonstrate effects that cannot be easily ruled out as post-perceptual. Nevertheless, we think these studies do not sufficiently meet the pre-perceptual challenge. Thanks for an anonymous reviewer for bringing these studies to our attention.

17 For defenses of the view that perceptual experience can have a very rich representational content see Siegel (2010) and Masrour (2011).

18 Of course, in such a case the label “visual module” may not be the best label anymore.

19 Thanks to an anonymous reviewer whose comment helped us clarify this point.

20 For evidence that deployment of spatial attention could influence subsequent perceptual categorizations see Kietzmann et al. (2011). Thanks to an anonymous reviewer for bringing this study to our attention.

21 Raftopoulos (2009) is an example of this strategy.

22 Some of the main proponents of the encapsulation thesis associate perceptual modules with a neuroanatomically demarcatable area of the brain. This is explicit in Fodor (1984) and Pylyshyn (1999) also seems to tacitly endorse it. Nevertheless, these theorists have not explicitly proposed any specific neuroanatomical demarcation of the boundaries of the module.

23 This, of course, under describes the complexity of the neural structures that underlie vision. For example, area MT receives connections from the retinal ganglion cells through the pulvinar structure in the thalamus and the superior colliculus (the tectum). These connections entirely bypass the LGN and the V1–V4 areas. So the place of area MT in the hierarchy is not completely clear. More importantly, the visual pathways also host an abundance of feedback connections that relay activity from higher areas of cortex to areas even as low as the retinal ganglion cells. So the idea that there is a simple neuroanatomical hierarchy in the visual system is somewhat questionable. Nevertheless, these complications can be accommodated within a general hierarchical framework that allows for multiple hierarchical schemes.

24 The distinction between the two visual streams is, of course, controversial (see Schenk and McIntosh, 2009, for a review).

25 For discussions of the debate concerning the circuitry of contextual effects (see Allman et al., 1985; Knierim and Van Essen, 1992; Lamme, 1995; Zipser et al., 1996; Nothdurft et al., 2000; Hupe et al., 2001; Jones et al., 2001; Angelucci et al., 2002; Cavanaugh et al., 2002; Levitt and Lund, 2002; Bair et al., 2003; Angelucci and Bressloff, 2006).

26 We will consider modifications of our working hypothesis that include adding MT to the module at the end of this section.

27 Raftopoulos (2006) also points out that according to ERP findings, some modulations due to spatial attention only affect V4 and not the previous areas.

28 See Raftopoulos (2009, 2014). Raftopoulos sometimes refers to this as the “rigging up” of the activities of these neurons.
